# The *Drosophila* Model: Exploring Novel Therapeutic Compounds against Neurodegenerative Diseases

**DOI:** 10.3390/antiox8120623

**Published:** 2019-12-06

**Authors:** Sun Joo Cha, Hyeon-Ah Do, Hyun-Jun Choi, Mihye Lee, Kiyoung Kim

**Affiliations:** 1 Soonchunhyang Institute of Medi-bio Science, Soonchunhyang University, Cheonan 31151, Korea; cktjswn92@sch.ac.kr (S.J.C.); chj5913@sch.ac.kr (H.-J.C.); mihyelee@sch.ac.kr (M.L.); 2 Department of Medical Biotechnology, Soonchunhyang University, Asan 31538, Korea; alsx8431@sch.ac.kr

**Keywords:** polyphenol, antioxidant, *Drosophila melanogaster*, neurodegenerative disease

## Abstract

Polyphenols are secondary metabolites of plants, fruits, and vegetables. They act as antioxidants against free radicals from UV light, pathogens, parasites, and oxidative stress. In *Drosophila* models, feeding with various polyphenols results in increased antioxidant capacity and prolonged lifespan. Therefore, dietary polyphenols have several health advantages for preventing many human diseases, including cardiovascular diseases, cancer, and neurodegenerative diseases. However, the exact role of polyphenols in neurodegenerative diseases is still yet to be completely defined. This review focuses on the most recent studies related to the therapeutic effect of polyphenols in neurodegenerative disease management and provides an overview of novel drug discovery from various polyphenols using the *Drosophila* model.

## 1. Introduction to Polyphenols

Plants, fruits, and vegetables store a lot of low molecular weight compounds that are secondary metabolites, and which are useful natural products to humans. One of the secondary metabolites is polyphenols. Many polyphenols display a protective effect against different kinds of stress, including reactive oxygen species (ROS), reactive nitrogen species (RNS), UV light, pathogens, parasites, and plant predators [1]. Therefore, many studies strongly recommend polyphenols for preventing cardiovascular disease, cancer, osteoporosis, and neurodegenerative disease [2]. Polyphenols are different compounds composed of one or more hydroxyl groups, which, based on the function or structural elements, are attached to one or more aromatic rings [3]. Accordingly, they are mainly categorized as flavonoids, phenolic acids, curcuminoids, stilbenes, and lignans (Table 1) [4,5].

Flavonoids are the largest group (60%) of dietary polyphenols [3]. The most common flavonoids are abundant in tea, various fruits, broccoli, onions, and soybeans [4,6]. They have a common carbon skeleton of diphenyl propane-flavone, two benzene rings, and a linear three carbon chain. The central three carbon chain may constitute a closed pyran ring with one of the benzene rings [6,7]. Most flavonoids are bound to sugars in the form of β-glycosides, and the sugar residue determines their absorption. Flavonoids are sub-divided into six classes depending on the oxidation state of the central pyran ring. In plants, ≥4000 flavonoids have been identified, and the numbers are increasing [8]. The biological function of flavonoids is antioxidative, effectively removing free radicals, like superoxide anions, and regulating enzyme activity induced by oxidative stress [9].

The second major group of polyphenols are phenolic acids, representing one-third of consumed phenolic compounds [10], and present in leguminous plants, broccoli, berry fruits, apples, coffee, tea, wine, and olive oil [2,5]. They are divided into two major classes, including hydroxybenzoic and hydroxycinnamic acid derivatives. They are presented as esters in foods and are resoluble in vacuoles or insoluble in the cell wall [10]. Phenolic acids are also antioxidative as free radical scavengers and chelators [3].

Curcuminoids, such as turmeric are abundant in Indian curries and Chinese medicine. These polyphenols are potent antioxidants, antiseptics for wound healing, and anti-inflammatory agents [11,12]. Therefore, curcumins are broadly used as food preservatives, food coloring agents, and drugs [10].

Resveratrol, a member of the stilbene family of polyphenols, is an antifungal molecule produced against pathogens by various plant species under stress conditions, such as UV radiation [13]. Resveratrol improves mitochondrial function and antioxidant properties in patients with neurodegenerative diseases [14,15].

Lignans are polyphenolic compounds formed by the dimerization of two coniferyl or sinapyl alcohol units [16,17]. They exist in soybeans, whole grains, fruits, and vegetables, and constitute plant cell walls [5]. Lignans are the phenolic components of olive oil, have high antioxidant activity, and inhibit lipid peroxidation [18].

## 2. Functions of Polyphenols in *Drosophila Melanogaster*

To study the potential use of polyphenols derived from plant products or plant extracts, many researchers use model organisms, such as mice, *Drosophila melanogaster*, zebrafish, *Caenorhabditis elegans*, and yeast [19]. This review focuses on in vivo *Drosophila* models used for investigating plant polyphenols. *D. melanogaster* has a number of advantages compared to mammalian models, including their short life cycle, small body size, easy genetic manipulation, large number of progenies, low maintenance costs, exhibition of complicated behaviors, and less rigid ethical concerns.

In flies, longevity is one of the phenotypes that is affected by feeding diets supplemented with many chemicals. By feeding larva with resveratrol, adult flies lived longer in a dose-dependent manner. This is similar to the longevity extension by *Aloe vera* extract supplementation, which is mediated via prevention of neurodegeneration or nerve fiber regeneration due to improved locomotor activities. Increased activity of the detoxifying enzymes, superoxide dismutase (SOD), and catalase, seems to cause damage in other ways and eliminate toxic elements that do not prolong life [20]. Moreover, longevity in both female and male flies was prolonged by dietary intake of pomegranate juice. Pomegranate has many known functions, including antioxidant, antiviral, anti-diarrhea, and lipid regulation. Pomegranate juice-fed flies showed improved climbing activity [21]. Apple polyphenols are an excellent source of dietary antioxidants and have been associated with antiaging. Apple is composed of several molecules, including catechin and chlorogenic acids, which have been identified by high-performance liquid chromatography (HPLC). In a fly model, supplementation of apple polyphenols in the diet dramatically prolonged lifespan compared with the control. Its antiaging activity is mediated by up-regulation of the antioxidant enzymes, SOD1, SOD2, and catalase, and down-regulation of the longevity determining gene, *Methuselah* (MTH) in aged flies. Dietary apple polyphenols partially recovered chronic paraquat exposure-induced fatality and weakened climbing mobility [22].

One of the most commonly consumed beverages and a complex of phenolic and non-phenolic antioxidant compounds is coffee. Using the sex-linked recessive lethal test, fewer lethal mutations were observed in the germ cells of larvae and adult flies treated with coffee and cyclophosphamide than those treated with cyclophosphamide alone. Additionally, the antioxidant activity of coffee is enhanced against cyclophosphamide-induced oxidative stress [23]. Caffeine is broadly consumed especially in coffee and teas, and has led to sleep problems, like sleep disturbance and daytime sleepiness in many adults [24]. The effect on the locomotive activity and behavioral patterns of a fly model of feeding roasted, explosion-puffed, or decaffeinated coffee beans was investigated. Explosion-puffed coffee had more antioxidants as well as chlorogenic acid, and enhanced sleep through γ-aminobutyric acid (GABA) and 5-hydroxytryptophan (5-HTP) [25]. Another globally popular drink is green tea, an attractive source of health-regulating dietary antioxidants [26]. In one study, feeding male flies with green tea extracts prolonged their lifespan and increased the antioxidant activities of SOD and catalase [27]. This was attributed to catechins, the most abundant polyphenols in green tea, which protected the flies from iron toxicity. Another study also attributed longevity to the inhibition of age-related iron accumulation [28]. Meanwhile, green tea has both positive and negative effects in flies; although the male flies fed green tea had a longer lifespan, their fertility declined simultaneously. Also, embryos of flies treated with green tea had significantly reduced offspring numbers, and larvae that successfully developed into adults were dramatically smaller in body size [26].

The various biological functions of dietary extracts of pure polyphenols, such as flavonoid and gallic acid (GA), have been demonstrated in the fly model. To ensure its effects, GA was fed to adult flies and larva. When fed to flies cotreated with urethane (URE), a genotoxic material, GA played a critical antigenotoxic role and enhanced antioxidant enzymes, such as glutathione S-transferase activity, SOD, and catalase. Feeding GA to adult and larva reduced the frequencies of sex-linked recessive lethal mutations at all germ cell stages. In flies treated with carcinogens and mutagens, dietary GA displayed antimutagenic effects at the pre- and post-meiotic germ cell stages [29]. The antioxidant activity of flavonoids and its advantages in human disease conditions have been well studied, but there little is known about modulating its effects on lipid droplet biogenesis. In the fly model, the effect of flavonoids diminishes fat storage, the number and size of lipid droplets, and total area occupied by lipid droplets. After supplementation of fly diet with flavonoids, the increase of lipid droplets in oenocytes indicated that fat body lipolysis was activated. The central nervous system and muscles require high energy, so the number of lipid droplets increased in glial cells and muscles after treatment with flavonoids. Therefore, the administration of flavonoids regulates lipid droplets biogenesis in a tissue-specific manner and consumption of flavonoids is a useful therapy for neuronal and metabolic diseases [30].

## 3. The Effects of Polyphenols on Neurodegenerative Diseases Using *Drosophila* Models

Many studies have demonstrated the role of polyphenols in the prevention of cardiovascular disease, cancer, osteoporosis, and neurodegenerative disease [2]. This review is focused on the effects of polyphenols in many neurodegenerative diseases.

Neurodegenerative diseases, including Alzheimer’s disease (AD), Parkinson’s disease (PD), Huntington’s disease (HD), and Amyotrophic lateral sclerosis (ALS), involve the progressive degeneration of structure or function, and death of neurons. The diseases have several common pathological and pathogenetic characteristics, such as specific protein aggregation, mitochondrial dysfunction, neuro-inflammation, and iron toxicity. Also, oxidative and nitrosative stresses are affected in the pathology of neurodegenerative disease [31,32]. However, the critical pathogenesis and therapeutics of neurodegenerative disease remains mostly unknown. Recently, many researchers have focused on diet therapy of neurodegenerative diseases for treatment. The neuroprotective effects of some polyphenol dietaries can be potentially useful for treatment or pharmacotherapy as suggested by several studies (Table 2). This review also discusses the potential therapeutic applications of polyphenols in neurodegenerative diseases using the *Drosophila* model.

### 3.1. Alzheimer’s Disease (AD)

Alzheimer’s disease (AD) is a prevalent and progressive chronic neurodegenerative disease. Decreasing verbal ability and motor neuron function and cognitive impairment are symptoms of AD. On average, AD patients live for 8 years post-diagnosis, depending on individual age and other health conditions [48,49]. This disease is related to a brain pathology that includes neurofibrillary tangles composed of hyperphosphorylated Tau protein and senile plaque formation composed of amyloid beta protein (Aβ) aggregates [50]. Tau plays an important role in modeling microtubules and bridging these polymers with other cytoskeletal filaments [51]. Since the tau protein becomes tangled in nerve cells, microtubules break up and destroy cellular structure. These events induce disintegration of the transportation system and miscommunication between neurons, and finally lead to cell death. Extracellular aggregates of amyloid plaques mostly consist of abnormally folded products of both amyloid precursor protein (APP) metabolism, Aβ40, and Aβ42 [52]. In the brain of AD patients, Aβ42 is highly amyloidogenic and accumulates more commonly than Aβ40 [48,52]. The number of AD patients is growing rapidly, and there are no effective treatments yet. That is why new drugs for AD treatment and prevention are needed. The use of plant-derived extracts and ingredients for the treatment and prevention of many types of diseases, including AD, is widely reported.

Among various plant-derived extracts or products for curing AD, grape-seed polyphenolic extract (GSPE) was used to prevent the abnormal oligomerization of Aβ in a mouse model of amyloid neuropathology [53,54], and to break down existing aggregated tau peptides in vitro [55]. Moreover, GSPE has a beneficial role in tau-mediated neuropathology in *Drosophila* models. Overexpressing *R406W* mutant tau in the fly eye results in a dramatic reduction and degeneration of the eye morphology. Among these phenotypes, *R406W* mutant tau is one of the modeled aspects of tauopathy in the *Drosophila* model [56]. GSPE therapy in flies overexpressing mutant tau in the eye results in the recovery of eye size and morphology. GSPE-mediated ameliorations in the *Drosophila* eye model likely take place following the manufacture of the toxic protein, but upstream of caspase activation [33].

Additionally, polyphenols in adzuki beans are known to inhibit the aggregation of various amyloid proteins [57,58]. Deposition of Aβ42 aggregates in the brain and oxidative stress are AD symptoms. Adzuki bean extract restored the memory abnormalities in Aβ42 overexpressing flies, originally caused by the suppression of Aβ42 aggregation and oxidative stress. Also, defects in mobility and the shortened lifespan of the fly model were restored by the adzuki bean extracts [34]. Thus, adzuki bean polyphenols may delay the progression and prevention of AD.

Since *Arabidopsis thaliana* grows rapidly, and has been fully sequenced, its extracts are widely used in research. These extracts might also be applicable in AD therapy, as they are rich in phenolic compounds useful against inflammation by activating the Nrf2 pathway in BV2 cells. *Drosophila* overexpressing the human Aβ42 peptide were used to confirm the activity of phenolic compounds in *Arabidopsis thaliana*. Supplementation of polyphenolic extracts restored the defective climbing ability of AD-induced flies [35].

Tobacco and coffee, containing nicotine or caffeine, provide the symptomatic alleviation that may lead to neuroprotection [59,60]. The neuroprotective impacts of coffee and tobacco were not derived from caffeine or nicotine in a PD model [36,59,61]. Decaffeinated coffee and nicotine-free tobacco restored defective phenotypes, including damaged climbing ability, reduced survival rate and declined eclosion rate in an AD fly model induced by Aβ42 overexpressed [36]. These studies suggest that decaffeinated coffee and nicotine-free tobacco confer significantly protective effects on the AD model.

### 3.2. Parkinson’s Disease (PD)

Parkinson’s disease (PD) is the second most common neurodegenerative disease after AD, and is characterized by progressive and selective loss of dopaminergic neurons from the substantia nigra region of the brain [62]. PD is grouped as sporadic or familial and is specific when the specific cause is unknown, but related to oxidative stress [63]. Genetic mutations in specific proteins, including PARK2 (parkin), SNCA (α-synuclein), PINK1 (pink1), and PARK7 (DJ-1), leads to familial PD [64,65,66]. One of the pathological hallmarks of PD is the formation of Lewy bodies, including the abnormal expression of α-synuclein, a presynaptic neuronal protein related to regulation of the dopamine and a major fibrillar component of Lewy bodies [67]. Under pathological conditions, α-synuclein becomes insoluble and forms toxic accumulations [68]. The exact cause of PD still remains poorly understood but oxidative stress plays an important role in neuronal decline [69]. Further, exposure to environmental toxins like MPTP (1-methyl-4-phenyl-1,2,3,6-tetrahydropyridine) and paraquat (PQ) induce severe and irreversible parkinsonism [70,71,72]. Since, PQ^2+^ has a similar chemical structure to MPP^+^, which is an active metabolite of MPTP, it is presently used in PD models [73]. Traditional herbal compounds with antioxidant properties are a proven source for therapeutic drug development for PD [74].

For example, the root extract of *Decalepis hamiltonii* (*Dh*) is known as a novel natural antioxidant. Dietary supplementation with *Dh* root extract antioxidants in flies overexpressing both missense mutations (A30P and A53T) of α-synuclein restored defective motility and circadian rhythm, as well as diminution of ROS and lipid peroxidation, and enhancement of catalase and SOD activities. Indeed, *Dh* extract reduced neurotoxicity against PQ sensitivity due to the mutations [37] and it can be used in PD therapy to defer the onset of PD.

Similarly to *Dh*, avocado *(Persea americana*), a fruit broadly cultivated in tropical and subtropical climates globally [75], is also a source of antioxidants for PD treatment. The Colinred peel (CRE) and epicatechin (EC) in methanolic *P. Americana* extracts protect *parkin* knockdown flies exposed to PQ by enhancing the lifespan and locomotor activity. Hence, its extract can protect *parkin* knockdown flies against PQ-induced oxidative stress, mobility damage, and lipid peroxidation [38].

Among the polyphenols from fruits, tangeritin, a flavonoid, found in the peels of Mandarin oranges, has various biological activities, such as neuroprotection, improving the gap junction intercellular communication, apoptosis, and antimetastasis [76,77,78,79]. The exposure of PD flies to tangeritin increased the dopamine content and restored the reduction in locomotive activity and various oxidative stress markers, such as lipid peroxidation, reduced glutathione, glutathione s-transferase, protein carbonyl content, and monoamine oxidase activity [39]. Therefore, supplementation of tangeritin led to a reduction in PD symptoms, suggesting its potential application in dietary therapy.

Grape and grape seed extracts, rich in polyphenols (flavonoids and GA), are well-known sources of antioxidants in neurodegenerative disease therapy [33,40,45]. When the extract was fed to flies expressing α-synuclein, female flies showed significantly expanded longevity and male flies showed highly enhanced climbing ability, confirming the ability of grape extracts to protect against free radicals and free radical-induced lipid peroxidation and DNA damages [40].

Treatment with capsaicin exerted a protective effect on PD flies induced with overexpressed α-synuclein, leading to delayed reduction in climbing ability. Supplementation with capsaicin is a potential agent for delaying PD development [41]

Due to its biological functions, such as antioxidation, anti-inflammation, and prevention of several diseases, tea drinking is popular worldwide. Black tea has a protective effect against PD symptoms in PD-induced flies using a model exposed to l-dopamine, by delaying the reduction in lipid peroxidation and protein carbonyl content, increasing glutathione and dopamine content, and reducing glutathione s-transferase activity in a dose-dependent manner [42].

As already mentioned, decaffeinated coffee and nicotine-free tobacco had neuroprotective effects in an AD fly model in a similar manner to that in the PD transgenic fly overexpressing the α-synuclein and loss-of-function *parkin* gene mutant. Decaffeinated coffee and nicotine-free tobacco have neuroprotective effects through the activation of the cytoprotective transcription factor Nrf2 [36]. Hence, these compounds serve as therapeutic candidates in AD and PD models.

Polyphenolic extracts, phenolic acids, and flavanols, have antioxidant activity and protective effects against PD-induced exposure to iron and PQ. Pure polyphenols, including GA, caffeic acid (CA), propyl gallate (PG), and epigallocatechin-3-gallate (EGCG), rescued the impaired climbing capability induced by PQ in the fly. PG and EGCG polyphenols in particular protected the locomotive capability of flies cotreated with PQ and iron [80]. Besides, curcumin exposure in α-synuclein overexpressing PD flies dramatically increased the life span and reduce oxidative stress, representing a reduction in lipid peroxidation and protein carbonyl content, and apoptosis [43]. GA significantly preserved the number of dopaminergic neurons, and improved life span and locomotive activities under PQ treatment [44]. Therefore, the various polyphenolic compounds discussed are potential sources for drug therapy of neurodegenerative disease.

### 3.3. Huntington’s Disease (HD)

Huntington’s disease is an autosomal neurodegenerative disease characterized by the degeneration of neurons and cognitive symptoms, which finally results in death. HD is caused by the elongation of a polymorphic CAG triplet repeat in the first exon of the *huntingtin* (*HTT*) gene that is translated to an elongated polyglutamine (polyQ) repeat in the mutant huntingtin (htt) protein [81,82]. Mutant htt aggregate formation by its N-terminal cleavage is implicated in HD toxicity, which leads to neuronal damage and loss [83]. The inclusion of mutant htt negatively affects intracellular processes acting as mitochondrial and transcriptional systems of genes, disturbed calcium signaling, aberrant protein–protein interactions, adjustments in the ubiquitin-proteasomal system, and autophagy [81]. Therefore, the inhibition of abnormal htt protein aggregate formation is a novel approach for HD therapy.

One of the dietary control treatments for HD is GSPE, like in AD [45]. Postmortem brains of HD patients showed increased oxidative damage [84,85], thus oxidative stress is a representative indicator of HD pathogenesis. GSPE could physically disrupt the aggregation of Aβ and Tau peptides [33,53,55]. Moreover, GSPE treatment dramatically prolonged lifespan in *Drosophila* and R6/2 mouse HD models and also rescued the motor defects in the mice [45].

Another HD treatment is green tea. Among the green tea polyphenols, EGCG blocks the mutant htt protein aggregates in a dose-dependent manner. In a yeast HD model, EGCG treatment dramatically down-regulated polyQ-mediated htt protein aggregation and cytotoxicity. Photoreceptor degradation and motor deficits were restored in transgenic HD flies overexpressing the pathogenic htt when fed with EGCG [46]. Although treatment with green tea did not change the reduced viability induced by mutant huntingtin, supplementation with green tea infusion mitigated the reduced lifespan and neurodegeneration caused by the mutant huntingtin in a *Drosophila* model [47]. Therefore, green tea consumption could become a reasonable HD therapy.

## 4. Conclusions

Here, we review plant-derived polyphenols that have shown several beneficial effects in *Drosophila* models of various neurodegenerative diseases (Table 2). Furthermore, we provide useful values and features of *Drosophila* to screen the new drugs for the patients of neurodegenerative diseases (Table 3). Despite considerable evidence on the relationship between polyphenol treatment and neuroprotection, the precise mechanism and usage for disease therapy remain elusive and limited. Comprehensive analyses of various experiments using *Drosophila* models of neurodegenerative diseases in combination with clinical studies and patient data should be carried out to help discover novel drugs that can improve defective neurodegenerative disease phenotypes.

## Figures and Tables

**Table 1:** Categorization and chemical structure of polyphenols.

Categories of Polyphenol	Basic Chemical Structure
Flavonoid	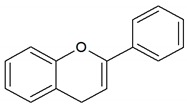
Phenolic acid	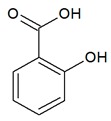
Curcuminoid	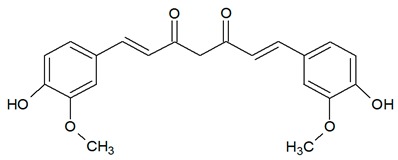
Stilbene	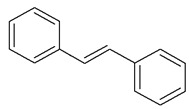
Lignan	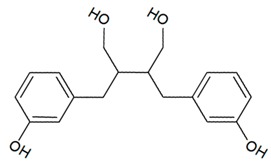

**Table 2:** Studies on the physiological function of polyphenols in neurodegenerative diseases.

Neurodegenerative Disease	Polyphenol Derived from Plants	Supplemental Amount	Effect	Reference
Alzheimer’s disease (AD)	Grape seed	2.8 μg/mL GSPE in fly medium	Recover the defective *Drosophila* eye induced by tauopathy using *R406W* mutant tau fly	[33]
	Adzuki bean	1 mg/mL adzuki extract in fly medium	Restore abnormal memory, movement defects, and shortened lifespan in Aβ42-overexpressing fly	[34]
	*Arabidopsis thaliana (At)*	40 μL/mL ethyl acetate extract of *At* in fly medium	Recover defective climbing capability in Aβ42-overexpressing fly	[35]
	Decaffeinated coffee and nicotine-free tobacco	0.15% decaffeinated coffee and 0.03% nicotine-free tobacco in fly medium	Rescue damaged locomotive activity, shortened longevity, and reduced eclosion rate via the activation of Nrf2 in AD fly induced by Aβ42 overexpression	[36]
Parkinson’s disease (PD)	*Decalepis hamiltonii* (*Dh*) root	0.1 or 0.5% aqueous extract of *Dh* in fly medium	Restore the defective locomotive activity and circadian rhythm due to missense mutations (A30P and A35T) in an α-synuclein-overexpressing fly and protect against PQ sensitivity	[37]
	Avocado *Persea americana (Pa)* peel	1 or 5 mg/mL methanol extract of *Pa* in fly medium	Protect *parkin* knockdown fly against paraquat (PQ)- induced oxidative stress, mobility damage, shortened longevity, and lipid peroxidation	[38]
	Tangeritin	5, 10, and 20 μM extract of tangeritin in fly medium	Rescue the reduction in locomotive activity and several oxidative stress markers and increase the dopamine content in PD fly model	[39]
	Grape	0.16–0.64 mg extract in 100 g of fly medium	Protect against free radicals, free radical-induced lipid peroxidation, and DNA damages and life extension in an α-synuclein-overexpressing fly model	[40]
	Capsaicin	0.1–1.0 μL/mL of capsaicin in fly medium	Delay the reduction in climbing activity in an α-synuclein-overexpressing fly model	[41]
	Black tea	20, 40, and 60 μM extract of black tea in fly medium	Reduce lipid peroxidation, protein carbonyl content, and glutathione S-transferase activity, and increase glutathione and dopamine content in fly exposed to L-dopamine	[42]
	Decaffeinated coffee and nicotine-free tobacco	0.15% decaffeinated coffee and 0.03% nicotine-free tobacco in fly medium	Restore the damaged climbing ability, shortened survival rate, and reduce dopaminergic neurons via Nrf2 activation in mutant α-synuclein-overexpressing fly, and loss-of-function mutant in *parkin* gene	[36]
	Curcumin	25, 50, and 100 μM curcumin in fly medium	Increase the life span and reduce apoptosis and oxidative stress, such as lipid peroxidation and protein carbonyl content, in α-synuclein-overexpressing fly model	[43]
	Gallic acid	0.1 mM gallic acid in fly medium	Rescue the loss of dopaminergic neurons and alleviate life span and locomotive activity with PQ treatment	[44]
Huntington’s disease (HD)	Grape seed	2.8 μg/mL GSPE in fly medium	Prolong the lifespan in Q93httexon1-overexpressing fly model	[45]
	Green tea	0.01–100 μM EGCG in fly medium and water extract of green tea in fly medium	Prolong the lifespan in Q93httexon1-overexpressing fly model	[46,47]

**Table 3:** Summary of the phenotypes in *Drosophila* models of neurodegenerative diseases.

Neurodegenerative Disease	Type	*Drosophila* Model	Phenotype	Symptoms of Human Patient	Reference
Alzheimer’s disease (AD)	Familial	Tau^R406W^-overexpression in the eye using an *ey-Gal4*	Rough eye, reduced locomotion, and shortened lifespan	Impairment of learning and memory, sleep disorder, motor impairment, and decreased attention	[33,34,35,36]
		Aβ42-overexpression in neurons using an *elav-Gal4*	Reduced locomotion and shortened lifespan		
		Aβ42-overexpression in glial cells using a *repo-Gal4*	Reduced locomotion, shortened lifespan, and defect in memory and learning		
Parkinson’s disease (PD)	Familial	α-synuclein^A30P^- and α-synuclein^A53P^-overexpression in neurons using an *elav-Gal4*	Reduced locomotion, shortened lifespan, increased lipid peroxidation, and reactive oxygen species	Tremor, rigidity, impaired balance, motor impairment, depression, and sleep disorder	[36,37,38,39,40,41,42,43,44]
		*Parkin* RNAi-overexpression in dopaminergic neurons using a *TH-Gal4*	Reduced locomotion and shortened lifespan		
		α-synuclein-overexpression in neurons using an *elav-Gal4*	Reduced locomotion, increased lipid peroxidation and reactive oxygen species, and decreased dopamine content		
		α-synuclein-overexpression in dopaminergic neurons using a *Ddc-Gal4*	Reduced locomotion and shortened lifespan		
		α-synuclein-overexpression in dopaminergic neurons using a *TH-Gal4*	Reduced locomotion and dopaminergic neuron loss		
		*parkin* null mutant	Dopaminergic neuron loss		
	Sporadic	Chronic exposure to paraquat	Reduced locomotion and shortened lifespan		
Huntington’s disease (HD)	Familial	Q93httexon1-ovexpression in neurons using an *elav-Gal4*	Reduced locomotion, shortened lifespan, and degeneration of photoreceptor neuron	Impairment of learning and memory, rigidity, and movement disorder	[45,46,47]
